# Trial sequential meta-analysis of laparoscopic versus open pancreaticoduodenectomy: is it the time to stop the randomization?

**DOI:** 10.1007/s00464-022-09660-6

**Published:** 2022-10-17

**Authors:** Claudio Ricci, Alberto Stocco, Carlo Ingaldi, Laura Alberici, Francesco Serbassi, Emilio De Raffele, Riccardo Casadei

**Affiliations:** 1 Division of Pancreatic Surgery, IRCCS Azienda Ospedaliero-Universitaria Di Bologna, via Albertoni 15-Italia, Bologna, Italy; 2 Department of Internal Medicine and Surgery (DIMEC), Chirurgia Generale-Minni, Alma Mater Studiorum-Università di Bologna, Policlinico S.Orsola-Malpighi Via Massarenti n.9, 40138 Bologna, Italy

## Abstract

**Background:**

The advantages of LPD compared with OPD remain debatable. The study aimed to compare the laparoscopic (LPD) versus open (OPD) for pancreaticoduodenectomy.

**Methods:**

A meta-analysis of randomized studies (RCTs) comparing LPD and OPD was made. The results were reported as relative risk (RRs) or mean differences (MDs). The trial sequential analysis was used to test the type I and type II errors defining the required information size (RIS). The primary outcome was mortality, major morbidity, and postoperative pancreatic fistula (POPF). R1 resection, post-pancreatectomy hemorrhage, delayed gastric emptying, biliary fistula, reoperation, readmission, operative time (OT), lymph nodes harvested, and length of stay (LOS) were also studied.

**Results:**

Four RCTs, counting 818 patients, were found. The RRs for mortality, major morbidity, and POPF were 1.16, 1.04, and 0.86, without significant differences. The RISs were 35,672, 16,548, and 8206. To confirm this equivalence, at least 34,854, 15,730, and 7338 should be randomized. OT was significantly longer in LPD than OPD, with an MD of 63.22. The LOS was significantly shorter in LPD than in OPD, with − 1.76 days. The RISs were 1297 and 1273, excluding a false-positive result. No significant differences were observed for the remaining endpoints, and RISs suggested that more than 3000 patients should be randomized to confirm the equivalence.

**Conclusion:**

The equivalence of LPD and OPD for mortality, major morbidity, and POPF is affected by type II error. The RISs to demonstrate a superiority of one of the two techniques seem unrealistic to obtain.

**Supplementary Information:**

The online version contains supplementary material available at 10.1007/s00464-022-09660-6.

Laparoscopic pancreaticoduodenectomy (LPD) remains a challenging surgical procedure, despite the recent advances in abdominal minimally invasive surgery [1]. However, even if the open approach (OPD) is the gold standard, the LDP has recently gained popularity, and Miami Guidelines1 suggested the minimally invasive technique could be considered a valid approach for selected patients with periampullary cancer. Two recent meta-analyses [2, 3] of randomized clinical trials (RCTs) have demonstrated no significant differences between the two approaches. Nonetheless, some doubts remained, and it seems crucial to assess if the non-significance is due to an absolute equivalence of the two techniques or lack of power of meta-analyses. In the first scenario, the scientific community should produce a further effort to demonstrate superiority or non-inferiority definitively, performing new RCTs if the required sample size is reasonable. In the second scenario, the adequate sample size is probably yet reached, and any additional RCT would be time consuming, expensive, and inefficient to demonstrate the superiority or non-inferiority of LPD. The trial sequential analysis (TSA) can be used to deal with these problems. TSA combines conventional meta-analysis methodology with an algorithm already used in the interim analyses in randomized clinical trials [4, 5]. Using the TSA approach, it is possible to include all RCTs available in chronological order and calculate the sample size necessary to accept or reject the statistical hypothesis “a priori.” TSA permits to evaluate if the results of the meta-analysis are correct, overestimated (type I error), or underestimated (type II error), [6, 7] assessing the number of patients required to obtain credible information.

The present study aims to perform an updated systematic review and a trial sequential meta-analysis, including all RCTs available comparing LPD versus OPD.

## Methods

The manuscript was prepared following the Preferred Reporting Items for Systematic Reviews and Meta-Analyses statement (PRISMA) [8]. The study is registered on PROSPERO with code number CRD42022299099. Information source, search, study selection, and data collection process are reported in Supplementary methods. The systematic review was conducted according to the recommendations from the Study Center of the German Society of Surgery [9].

### Eligibility criteria, items, and risk of bias in individual studies

The criteria were established according to the PICOS methodology [10]: the population was represented by all patients undergoing PD for benign or malignant pancreatic head lesions; the intervention arm was the LPD; and the control arm was OPD. All studies reporting postoperative results were included. Only RCTs were considered. The following information was extracted to define each study: authors, affiliation and country, year of publication, acronyms if present, registration number, the presence of blinded evaluation of outcomes, the learning curve of the surgeon involved, and the sample size. Post hoc analyses were added concerning the registered protocol: (i) postoperative pancreatic fistula (POPF), according to the new definition [11], was also considered a primary endpoint; (ii) also post-pancreatectomy hemorrhage (PPH) [12] delayed gastric emptying (DGE) grade B and C [13] were considered only when clinically relevant (grade B and C); and (iii) operative time was included among the secondary endpoints. Thus, the primary endpoints were 90-day mortality, major morbidity defined as Clavien–Dindo class III or higher [14], and POPF. As secondary endpoints, R1 resection rate, PPH and DGE grade B and C, biliary fistula, reoperation, readmission, operative time, lymph nodes harvested, and length of stay (LOS) were studied. After a collegial discussion involving the reviewers and the last author (R.C.), any disagreement was solved.

The qualitative assessment of the studies was carried out based on a revised tool for assessing the risk of bias in randomized trials (RoB-2) [15]. All variables were reported as frequencies and percentages or means and standard deviations (SD). The mean and SD were obtained using a dedicated statistical algorithm when the authors reported medians and interquartile or ranges [16, 17].

### Summary measures and synthesis of results

The TSA meta-analysis was performed to obtain the Risk Ratio (RR), mean difference (MD), and required information size (RIS). RRs and MDs are used for dichotomic and continuous outcomes, respectively, and they were reported together with a 95% confidence interval (95% CI). RIS represents the “a priori” sample size that should be collected to obtain credible results avoiding type I (false-positive results) and type II (false-negative results) [4]. RIS is calculated, taking into account the heterogeneity among the included studies, and the type I error was set at 5% and type II at 20% (power 80%) [5]. RIS was calculated using meta-analytical values of RRs and MDs for all endpoints, considering the heterogeneity. The RIS was also graphically reported in the Cartesian plane. *Y*-axis is the *Z*-score which corresponds to the conventional *P*-value. When the absolute value of the *Z*-score is higher than 1.96, *P*-value is less than 0.05, and the intervention effect is considered significant for classical meta-analysis. The *X*-axis represents the number of patients yet randomized, called “accrued sample size.” The *Z*-curve is obtained, adding each included trial sequentially. The *Z*-curve can cross three boundaries: the conventional (dotted red horizontal lines), monitoring boundaries (dotted black logarithmic lines), and futility boundaries (dotted black lines). The conventional edge corresponds to the nominal *P* = 0.05. False-positive results (type I error) are observed when the *Z*curve crosses this limit, but RIS is not reached. Conversely, the monitoring boundaries represent the values of *Z*-scores at which type I error is excluded. Indeed, when *Z*-curve crosses both conventional and monitoring boundaries, the significant results are credible, and no further randomization is needed to demonstrate one arm’s superiority. A false-negative effect (type II error) can be hypothesized when the *Z*-curve does not cross conventional and monitoring boundaries, but RIS is not reached [6–8]. However, if no effect is observed but the RIS is reached, type II error can be excluded. In this case, the *Z*-score crosses the futility boundaries, namely the threshold for non-superiority and non-inferiority. Any additional randomization is useless to show differences between the two arms. The Supplementary Fig. 1 explains the possible outputs based on the *Z*-curve route. Additional RIS values were calculated hypothesizing different aims: (a) to demonstrate a clear superiority of laparoscopic or laparotomic approach assuming a 50% relative risk reduction (RRR) favoring LPD or OPD, respectively, and (b) to demonstrate relative advantages for mini-invasive or open approach, assuming a 25% relative risk reduction in favor of LPD or OPD, respectively. The same four aims were created using credible MD values for operative time, harvested lymph nodes, and LOS. However, for operative time and lymph nodes gathered, the RISs were calculated to show the non-inferiority of LPD, the alternative and null hypothesis for harvested lymph nodes, and operative time was planned as a non-inferiority study because it seems unrealistic that LPD could be superior of OPD. The meta-analysis was carried out in line with recommendations from the Cochrane Collaboration [18], and the Mantel–Haenszel random-effects model was used to calculate effect sizes [19].

### Risk of bias across studies and meta-regression analysis

The heterogeneity was evaluated using *I*^2^ and Cochran’s *Q* statistics [20]. The heterogeneity was also calculated as diversity (*D*^2^) [21]. The effect of confounding covariates with a meta-regression analysis [22, 23]. The publication bias was evaluated using the Begg and the Egger tests [24], and a *P*-value < 0.05 indicated a non-negligible “small-study effect.” The statistical analysis was carried out using dedicated packages for STATA v14®. TSA was conducted using the Trial Sequential Analysis software [5]. The funnel plot was produced using Revman version 6.1.

## Results

### Studies selection, characteristics, and risk of bias within the studies

**Fig. 1:**
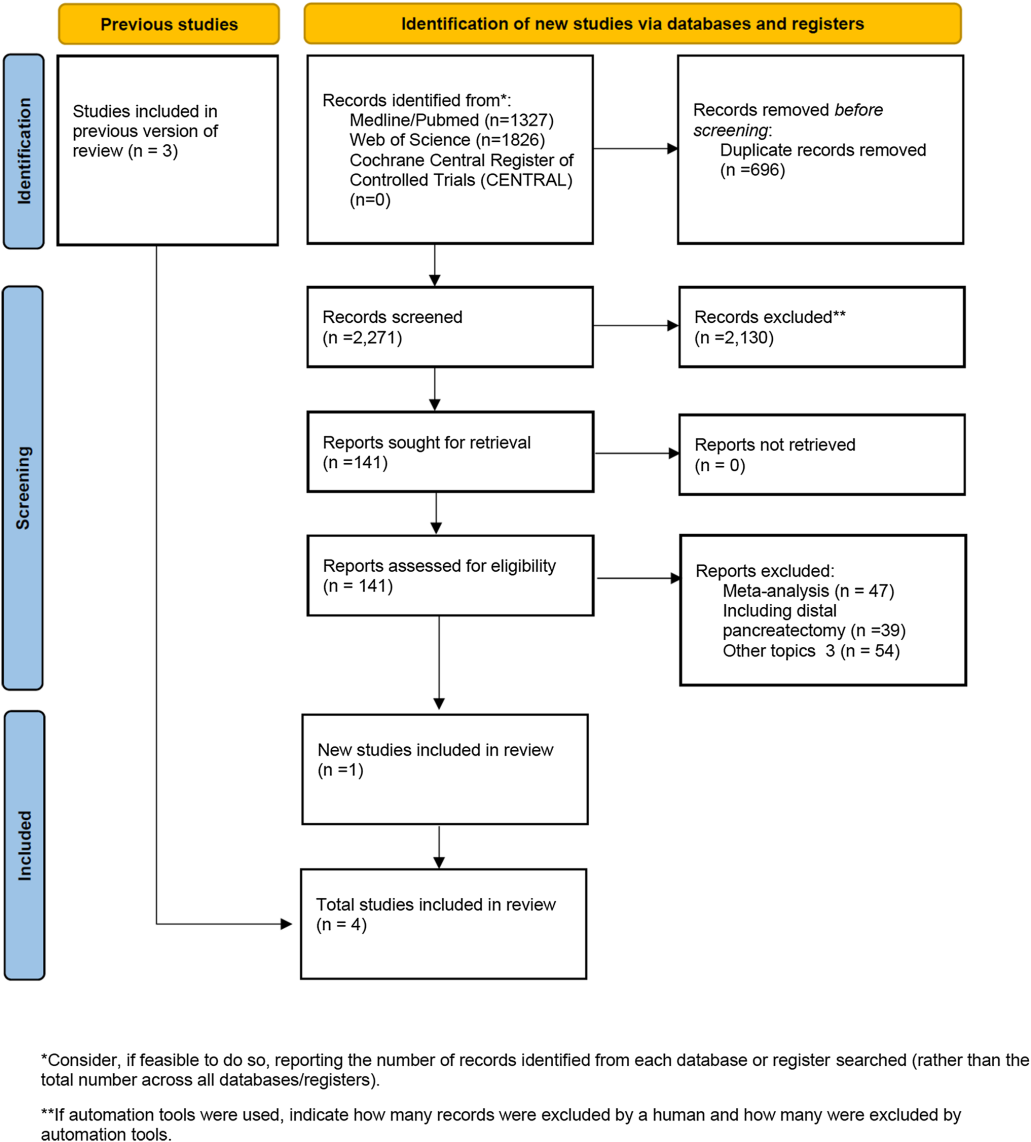
Flow diagram according to the PRISMA guidelines

PRISMA flowchart is reported in Fig. 1. The updated systematic search identified 3153 potential articles: 1327 from the Medline/PubMed database, 1826 from the ISI Web of Science, and 0 from CENTRAL. Two thousand two hundred seventy-one papers remained after de-duplication. Of these, 2130 were excluded because they were not pertinent to the study question. One hundred forty-one articles were reviewed in full-text form, and, of these, 140 were excluded. Finally, four trials [25–28] were available for analysis. There was 100% agreement between the two reviewers. The characteristics of selected studies are summarized in Table 1. The accrued sample size was 818: 411 (50.2%) in LPD and 407 (49.8%) in OPD. The differences between the two groups are described in Supplementary Table 1.

**Table 1:** Characteristics of the studies included

Authors	Affiliation/hospital	Year	Acronyms	Registration	Blinded	Pancreatic surgeon (*N*)	Learning curve in LPD (*N*; *N* of LPD)	RoB-2	Sample size
Palanivelu et al. [24]	Department of Surgical Gastroenterology and Hepatopancreatobiliary Surgery, GEM Hospital and Research Center, Tamil Nadu, India	2016	PLOT	NCT02081131	No	Yes (2)	Yes (2; > 25)	Some concerns	64
Poves et al. [25]	Department of Surgery, Hospital del Mar, Barcelona, Spain	2018	PADULAP	ISRCTN93168938	No	Yes (2)	Yes (1;–)	Some concerns	61
van Hilst et al. [26]	The Netherlands, multicenter	2019	LEOPARD-2	NTR5689	Patients-blinded	Yes (9)	Yes (9; > 20)	Low	99
Wang et al. [27]	China, multicenter	2021	TJDBPS01	NCT03138213	Patients-blinded	Yes (14)	Yes (14; > 104)	Some concerns	594
Total									818

*N* number, *LPD* laparoscopic pancreaticoduodenectomy, *RoB-2* A revised Cochrane risk-of-bias tool for randomized trials

### Results of individual studies and synthesis of the results

The results are reported in Table 2, while the additional RISs are reported in Table 3. All secondary endpoints are described in Supplementary results.

**Table 2:** Meta-analysis of all outcomes

Outcomes of interest	No. of studies	Event rate (%) or mean (SD)		RR or MD (95% CI)	*P* value	RIS	Δ	C-Q, I [2] (%), D (%)	*P* value for reporting bias ^	
		LPD arm	OPD arm						Egger	Begg
Primary endpoints										
90-day mortality	4	13/411 (3.2)	10/407(2.5)	1.16 (0.32 to 4.24)	0.542	35,672	− 34,854	0.194; 36; 50	0.642	1.000
Major morbidity	4	118/411 (28.7)	103/407 (25.3)	1.04 (0.70 to 1.54)	0.840	16,548	− 15,730	0.140; 46; 68	0.341	0.308
POPF	4	49/411 (11.9)	57/407 (14)	0.86 (0.60 to 0.02)	0.375	8,206	− 7388	0.403; 0; 0	0.393	0.734
Secondary endpoints										
Operative time (minutes)	4	338 ± 153	326 ± 75	63.22 (9.54 to 116.89)	0.020	1,273	− 455	< 0.001; 95; 99	–	–
PPH grade B and C°	4	34/411 (8.3)	40/407 (9.8)	0.84 (0.54 to 1.30)	0.438	11,486	− 10,668	0.740; 0; 0	0.391	0.734
DGE grade B and C°	4	63/411 (15.3)	65/407 (15.9)	0.95 (0.59 to 1.53)	0.820	253,461	− 252,643	0.170; 39; 59	0.713	0.308
Biliary fistula	4	25/411 (6.1)	19/407 (4.7)	1.33 (0.73 to 2.41)	0.350	8,183	− 7365	0.554; 0; 0	0.557	0.734
Reoperation	4	18/411 (4.3)	20/407 (4.9)	0.92 (0.42 to 2.01)	0.840	47,178	− 46,360	0.310; 17; 19	0.699	1.000
Readmission	4	27/411 (6.5)	23/407 (5.6)	1.12 (0.66 to 1.90)	0.680	22,032	− 21,214	0.568; 0; 0	0.784	0.734
LOS (days)	4	17 ± 12	20 ± 14	− 1.76 (− 3.32 to − 0.21)	0.030	1,297	− 479	0.530; 0; 0	–	–
Lymph node harvested	4	12 ± 5	13 ± 7	− 0.98 (− 2.40 to 0.44)	0.180	3,515	− 2693	0.008; 75; 91	–	–
R1 resection	4	26/411 (6.3)	33/407(8.1)	0.77 (0.50 to 1.19)	0.240	6,475	− 5657	0.940; 0; 0	0.243	0.308

*LPD* Laparoscopic pancreaticoduodenectomy, *OPD* Open pancreaticoduodenectomy, *SD* standard deviation, *RR* risk ratio, *MD* mean difference, *C–Q* *P* value of Cochrane’s test, *I*^2^ Higgins test, *D*^2^ diversity; ^A reporting bias non-negligible is considered for *P* values < 0.10; *POPF* clinical relevant postoperative pancreatic Fistula, *PPH* post-pancreatectomy hemorrhage according to ISGPS classification; *DGE* delayed gastric emptying according to ISGPS classification, *LOS* length of stay; –  not applicable; Null hypothesis (H0): LPD guarantees similar results concerning OPD; Alternative hypothesis (H1): OPD and LPD have different results; Power = this data is the probability of rejecting a false null hypothesis (H0); the pre-specified target value is 0.80; Alpha = It is the probability of rejecting a true null hypothesis; the pre-specified target value is 0.05. For dichotomic endpoint, we use a different level of Relative Risk Reduction (RRR), starting from the meta-analytic value; for the continuous value, we use an extra level of mean difference (MD), starting from meta-analytic value

**Table 3:** Additional RIS calculated hypothesizing different scenarios in planning new RCTs comparing LPD versus OPD

Parameters	Requirement Information Size			
	Best scenario in favor LPD (RRR 50%)	Intermediate scenario in favor LPD (RRR = 25%)	Best scenario in favor OPD (RRI = 50%)	Intermediate scenario in favor OPD (RRI = 25%)
Primary endpoints				
90-day mortality	7433	35,177	12,231	44,850
Major morbidity	933	4188	1313	4949
POPF	603	2755	926	3401
Secondary endpoints				
PPH grades B and C	891	4101	1406	5131
DGE grades B and C	1152	5269	1748	6427
Biliary fistula	1934	8887	3144	11,297
Reoperation	2282	10,492	3708	13,538
Readmission	1612	7465	2607	9456
R1 resection	1093	5069	1742	6371

Parameters	Requirement information size			
	Best scenario in favor LPD (MD + 10) ^^^	Intermediate scenario in favor LPD (MD = + 30) ^^^	Best scenario in favor OPD (MD + 120)	Intermediate scenario in favor OPD (MD = + 90)
Operative time	39,974	4269	374	599

Parameters	Requirement Information Size			
	Best scenario in favor LPD (MD -3) ^^^	Intermediate scenario in favor LPD (MD -1) ^^^	Best scenario in favor OPD (MD 3)	Best scenario in favor LPD (MD 1)
Lymph nodes harvested	302	2660	302	2660

Parameters	Requirement Information Size			
	Best scenario in favor LPD (MD -3)	Intermediate scenario in favor LPD (MD -1)	Best scenario in favor OPD (MD -5)	Intermediate scenario in favor LPD (MD -3)
LOS	354	3180	354	3180

*LPD* Laparoscopic pancreaticoduodenectomy; *OPD* Open pancreaticoduodenectomy, *RRR* relative risk reduction**, ***MD* mean difference, *POPF* clinical relevant postoperative pancreatic fistula, *PPH* post-pancreatectomy hemorrhage according to ISGPS classification, *DGE* delayed gastric emptying according to ISGPS classification, *LOS* length of stay; –not applicable; Null hypothesis (H0): *LPD* guarantees similar results concerning OPD; Alternative hypothesis (H1): OPD and LPD have different effects: in intermediate scenarios, the RRR was pre-set to 25% while in best methods was pre-set to 50%. For continuous value, the mean difference (MD) is set on credible values. Power = this data is the probability of rejecting a false null hypothesis (H0); the pre-specified target value is 0.80; Alpha = It is the probability of rejecting a true null hypothesis; the pre-specified target value is 0.05. ^the alternative and null hypotheses for harvested lymph nodes and operative time were planned as a non-inferiority study because it seems unrealistic that LPD could be superior to OPD.

### Primary endpoints

#### 90-day mortality

**Fig. 2:**
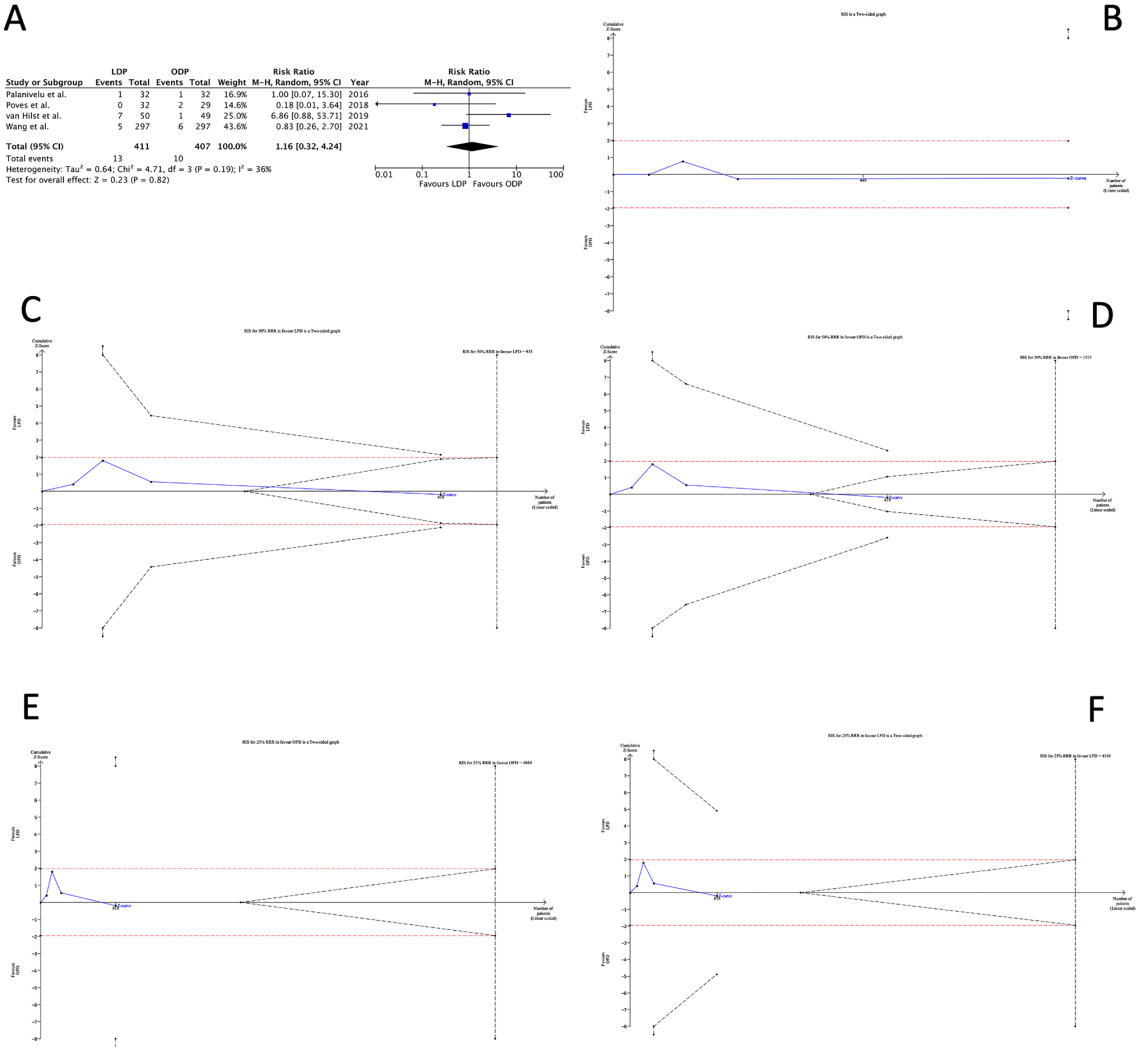
90-day mortality. Legend: **A** Forest plot; **B**–**F** the x-axis is the number of patients yet randomized; the y-axis is the cumulative z-score value representing the effect of each arm; and the blue line is the cumulative z-score obtained cumulating the studies. The dotted red horizontal lines are the conventional boundaries (p-value < 0.05). When z-curve crosses the conventional boundaries and the required information size (RIS) is not reached, the result is a false positive (“type I error”). When z-curve does not cross the conventional boundaries and RIS is not reached, the result is a false negative (type II error). The dotted black near-logarithmic lines are the monitoring boundaries. When the z-curve crosses the monitoring boundaries, the result is a true positive. The inverse dotted black lines are the futility boundaries (area in which any further randomization is useful). **B** At current RR equal to 1.16; **C** assuming that LPD could decrease by 50% the RR of mortality rates; **D** assuming that OPD could reduce by 50% the RR of mortality rates; **E** bearing that LPD could decrease by 25% the RR of mortality rates; and **F** assuming that OPD could reduce by 25% the RR of mortality rates

The risk of 90-day mortality (Fig. 2A) was similar among the two groups, with a pooled RR of 1.16 (0.32 to 4.24, 95% CI). The RIS at the current RR was 35,672, suggesting that 34,854 patients should be further randomized before concluding that LPD and OPD are equal without occurring in type II error (Fig. 2B). The closest horizon is 7433 patients, representing the number of patients required to exclude or demonstrate that LPD reduced mortality risk by 50%. A similar RIS (12,231) value was obtained, assuming that OPD could decrease by 50% the RR of mortality rates (Fig. 2C, D). The RISs required to get credible information for a 25% mortality reduction were 35,177 and 44,850 for laparoscopic and open approaches (Fig. 2E, F).

### Major morbidity (CDC > II)

**Fig. 3:**
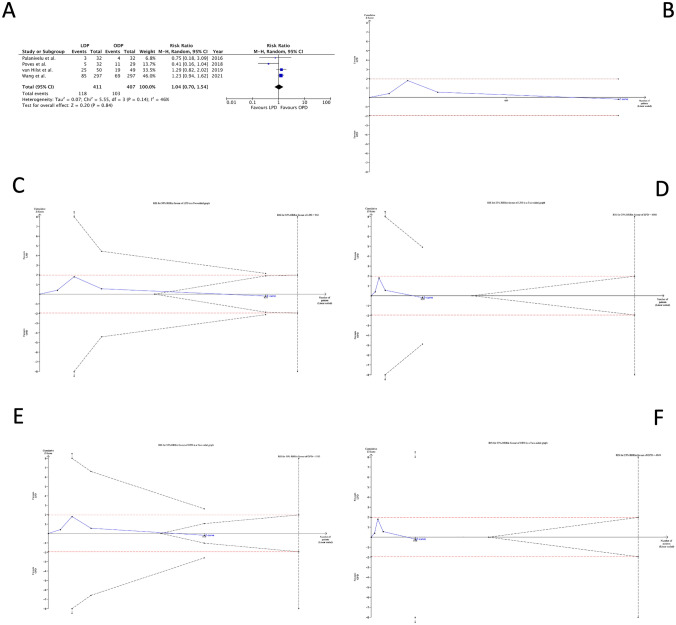
Major morbidity. **A** Forest plot of meta-analysis; **B–F** the *x*-axis is the number of patients yet randomized; the *y*-axis is the cumulative *z*-score value representing the effect of each arm; and the blue line is the cumulative *z*-score obtained cumulating the studies. The dotted red horizontal lines are the conventional boundaries (*p*-value < 0.05). When the *z*-curve crosses the conventional boundaries and the required information size (RIS) is not reached, the result is a false positive (“type I error”). When z-curve does not cross the conventional boundaries and RIS is not reached, the result is a false negative (type II error). The dotted black near-logarithmic lines are the monitoring boundaries. When the z-curve crosses the monitoring boundaries, the result is a true positive. The inverse dotted black lines are the futility boundaries (area in which any further randomization is useful). **B** at current RR equal to 1.04; **C** assuming that LPD could decrease by 50% the RR of major morbidity rates; **D** assuming that OPD could reduce by 50% the RR of major morbidity rates; **E** bearing that LPD could decrease by 25% the RR of major morbidity rates; and **F** assuming that OPD could reduce by 25% the RR of major morbidity

The risk of major morbidity (Fig. 3 A) was similar among the two groups, with an RR of 1.04 (0.70 to 1.54, 95% CI). At the current RR, the RIS required to reject the null hypothesis without type II error was 16,548, indicating that 15,730 should be additionally randomized (Fig. 3B). The additional RISs calculated for the four scenarios demonstrated that LPD and OPD did not reduce by 50% the risk of major complications because the Z-curve is close to RIS (933 and 1313). Still, it has crossed the futility boundaries (Fig. 3C, D). The assumption that LPD or OPD reduced by 25% of the RR could be demonstrated or rejected only cumulating 4188 or 4949 patients randomized (Fig. 3E, F).

#### POPF

**Fig. 4:**
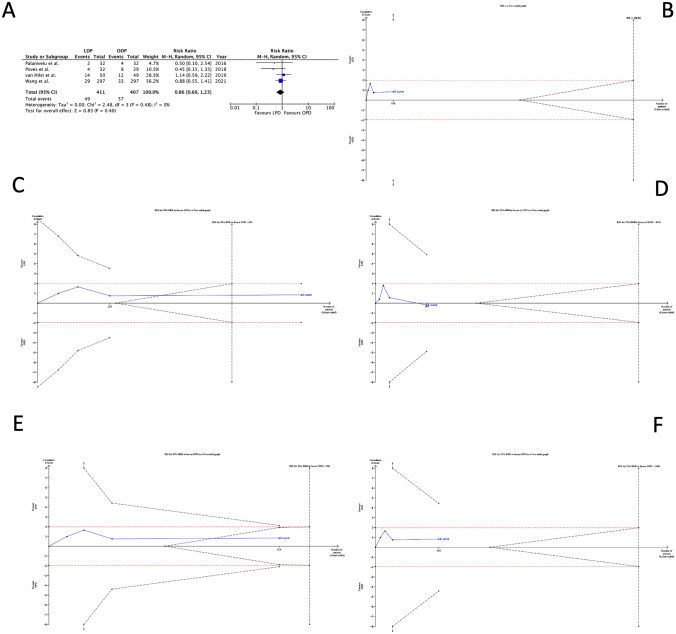
Clinically relevant postoperative pancreatic fistula. **A** Forest plot of meta-analysis; **B–F** the *x*-axis is the number of patients yet randomized; the *y*-axis is the cumulative *z*-score value representing the effect of each arm; and the blue line is the cumulative *z*-score obtained cumulating the studies. The dotted red horizontal lines are the conventional boundaries (*p*-value < 0.05). When *z*-curve crosses the conventional boundaries and the required information size (RIS) is not reached, the result is a false positive (“type I error”). When *z*-curve does not cross the conventional boundaries and RIS is not reached, the result is a false negative (type II error). The dotted black near-logarithmic lines are the monitoring boundaries. When the *z*-curve crosses the monitoring boundaries, the result is a true positive. The inverse dotted black lines are the futility boundaries (area in which any further randomization is useful). **B** at current RR equal to 1.04; **C** assuming that LPD could decrease by 50% the RR of clinically relevant postoperative pancreatic fistula rate; **D** assuming that OPD could drop by 50% the RR of clinically relevant postoperative pancreatic fistula rate; **E** bearing that LPD could reduce by 25% the RR of clinically relevant postoperative pancreatic fistula rate; and **F** assuming that OPD could decrease by 25% the RR of clinically relevant postoperative pancreatic fistula rate

The risk of POPF (Fig. 4A) was similar among the two groups, with a RR of 0.86 (0.60 to 0.02; 95% CI) and a RIS of 8026 (Fig. 4B). Additional 7338 patients should be randomized before accepting or rejecting the equivalence hypothesis of the two approaches. The additional RISs calculated for the four scenarios demonstrated that both LPD and OPD did not reduce by 50% the risk of POPF because the *Z*-curve is close to RIS (603 and 926). Still, it has crossed the futility boundaries (Fig. 4C, D). The assumption that LPD or OPD reduced by 25% of the RR could be demonstrated or rejected only cumulating 2755 or 3401 patients randomized (Fig. 4E, F).

### Heterogeneity, meta-regression analysis, and publication bias

A significant heterogeneity was observed for 90-day mortality (*I*^2^ = 36%, *D* = 50%), major morbidity (*I*^2^ = 46%, *D* = 68%), DGE grade B and C (*I*^2^ = 39%, *D* = 59%), operative time (*I*^2^ = 95%, *D* = 99%), and lymph node harvested (*I*^2^ = 75%, *D* = 91%). The RR of 90-day mortality increased in the LPD arm when the proportion of malignant lesions rose (+ 38.2; *P* = 0.035). Moreover, the RR of 90-day mortality increased in the LPD arm when the mean number per capita of procedures was superior to 20 (− 6.1; *P* = 0.035). The mean difference (minutes) in operative time seems shorter in patients with preoperative stent (− 391.4; *P* = 0.031) and those with the soft pancreas (− 147.1; *P* = 0.036). No covariates influenced the major morbidity rate, DGE, and harvested lymph nodes. No reporting bias was observed (Supplementary Tables 2–5).

## Discussion

The present study showed that the clinical safety of LPD, compared to OPD, is far from being proven and hardly demonstrable. To our knowledge, this systematic review represented the largest available, including four RCTs with an overall sample size of 818 patients: 411 (50.2%) in LPD and 407 (49.7%) in the OPD arm. The methodology is original because it permits including the studies in chronological order and not in “one-shot.” Moreover, TSA allows evaluating if the effects are credible or “at-risk” for false-positive and negative results compared with classical meta-analysis. The RRs of clinical safety indicators, such as 90-day mortality, major morbidity, and POPF, were similar among the two groups, suggesting the equivalence between the two approaches. The TSA demonstrated that this equivalence should be interpreted as a type II error. Indeed, several patients should be further randomized to accept the “null hypothesis,” namely that LPD guarantees similar mortality, major morbidity, and POPF to OPD. The required sample size was far from the accrued one, and this gap seems to be meant to remain large for several decades. Only 6 additional ongoing trials [29] were found on clinicaltrial.gov, counting a further 876 eligible patients to add to 818 available. The completeness of our search was confirmed by the paper of Probst et al. [30] that designed a detailed map of RCTs in pancreatic surgery. However, it should be noted that, regarding the mortality rate, this large RIS could be acceptable and irrelevant because this parameter is almost never used to calculate the sample size due to its rarity. On the contrary, the major morbidity or POPF rates are frequently used to sample size calculation for the new studies in minimally invasive pancreatic surgery. Therefore, despite the efforts made by the scientific community in planning, organizing, and conducting new RCTs, the sample size reachable in the following years seems to be insufficient. This observation opens an alarming and unexpected scenario: maintaining the current RRs between LPD and OPD, the sample size required to demonstrate credible results is too high and probably impossible to obtain, at least in a reasonable timeframe. Obviously, this does not mean that RCTs should be avoided in favor of low-quality studies. These results suggested that mini-invasive safety is challenging to demonstrate with RCT because the difference with OPD is minimal. In other words, these data tell us to avoid not the “useless RCTs” but the RCTs having “useless” endpoints, such as morbidity, mortality, or POPF.

Moreover, the uselessness of safety endpoints is even more disturbing, considering the difficulty of training in minimally invasive PD. The LPD required a learning curve, and incomplete training could produce poor results, as shown in the LEOPARD-2 trial by the Dutch Pancreatic Cancer Group [27]. On the contrary, the paper of Wang et al. [28] confirmed that the differences between the two approaches could be minimal in the presence of skilled surgeons. However, the learning curve seems unrealistic also for several high-volume surgical centers, requiring nearly 100 procedures for surgeons [31]. Moreover, a recent paper seems to demonstrate that morbidity and POPF rates decrease later than the operative time, only when the surgeon has completed the second phase of the learning curve for LPD (proficiency) [32]. Our meta-regression analysis also confirms these results: in trials where the surgeon performed less than 20 procedures, the risk of major morbidity increased in the LPD arm. If the data available did not clarify the safety of LPD, some helpful information could be extracted. Firstly, further randomization is useless to demonstrate that LPD or OPD could reduce the mortality, complication rate, or POPF by an RRR of 50%, showing that only benefits with low magnitude should be expected from one or other approaches. Practically, if the pancreatic surgeons could have high expectations in RCTs to demonstrate an impressive success or failure of LPD, they would be disappointed in its unprovability. Secondly, to accept or reject a marginal advantage, such as RRR by 25% of the mortality, major complication, or POPF, in any case, the number of patients required is hardly reachable in a reasonable period. The analysis of secondary endpoints confirmed similar results. PPH, DGE, biliary fistula, reoperation, readmission, mean lymph nodes harvested, and R1 resection rate are identical among the two groups. Some exciting findings should be observed by applying the TSA to operative time and LOS. The LPD is significantly longer than OPD, with an MD of one hour, and this result is closest to the benefit boundary in favor of OPD. On the contrary, the LOS is significantly shorter in LPD than in OPD by nearly two days. It is possible to consider these results credible and “not a risk” to type I error because the RIS is reached. However, these fascinating data impose some reflections. Without demonstrable clinical safety, the LPD should be accepted as “a new standard of care” only for undebatable clinical advantages. Nonetheless, the only benefit of LPD seems to be one or two days less in LOS. This parameter is a weak indicator of efficacy, easily influenced by patients’ and surgeons’ subjective perceptions, type of health care system, and availability of home program rehabilitation. Indeed, the difference between the two approaches disappeared when the LOS measurement was standardized using the functional recovery and the blind for the patients, such as in LEOPARD-2 [28]. On the contrary, in Wang et al. [31], despite a similar strategy to standardize the LOS measurement, different results in favor of LPD are observed. However, this study suffers from a critical bias. The discharge criteria are unbalanced in favor of the LPD approach because the authors precluded the discharge of patients with incision site infection. Surgical site infections are more frequent in laparotomic than laparoscopic procedures, commonly managed in an outpatient setting, and an unreasonable cause of prolonged hospitalization. Thus, the result seems to reflect a bias in the study’s design more than an actual efficacy of the minimally invasive approach. Once again, the results tell us that RCTs with the useless indicator of efficacy, such as operative time or LOS, should be further designed because they will not be informative. RCTs should not be avoided, but different outcomes could be studied, such as quality of life, costs from the health care system point of view, or both [32]. Otherwise, a different minimally invasive approach should be tested in RCTs. Indeed, a recent network meta-analysis suggests that robotic technology could be the best approach for PD among the minimally invasive available [33].

This study had some limitations. Firstly, even if only RCTs were considered, the quality of studies remained limited because none of the studies had blinded personnel and only two had blinded patients. Thus, although well defined in a standardized way, the recording and analysis of several endpoints could be influenced (e.g., LOS, operative time). Secondly, the study of some outcomes is affected by heterogeneity, such as 90-day mortality, major morbidity, operative time, DGE, and lymph node harvested. Meta-regression failed almost always in capturing the reason for the heterogeneity. Nonetheless, if the uninterpreted heterogeneity could suggest prudence in accepting positive and negative results, it is crucial to calculate the RIS correctly. Indeed, the TSA algorithm weights the presence of heterogeneity and considers this parameter to estimate the RIS prudentially.

In conclusion, our study did not recommend avoiding RCTs to explore the safety, feasibility, and efficacy of minimally invasive PD, but it underlined the risk of using “useless” endpoints to design further studies. The data suggested that LPD seems to provide marginal and debatable benefits compared to the actual gold standard, namely OPD. On the other hand, the learning curve appears to be extended, full of pitfalls, and within reach of a few high-volume centers. The results of six planned and ongoing RCTs available in future seem to be meant not to change the current state of things. Indeed, some certainties are yet available, and they will not change: the LPD requires a longer operative time than OPD and LPD could guarantee a slightly shorter hospitalization. Other results, such as equivalence in postoperative complications, including mortality, probably will never demonstrate due to the high number of patients required. Thus, different outcomes should be considered, such as quality of life and costs, or different minimally invasive techniques such as robotic ones should be considered.

Henceforth, the enrollment of further patients in studies evaluating LPD should be carefully assessed. If the study’s aim is to demonstrate the equivalence or superiority of OPD in the short-term outcomes, the randomization could be useless and time consuming.

## Supplementary Information

Below is the link to the electronic supplementary material.

Electronic supplementary material 1 (PNG 150 kb)

Electronic supplementary material 2 (DOCX 18 kb)

Electronic supplementary material 3 (DOCX 15 kb)

Electronic supplementary material 4 (DOCX 36 kb)
