# Supplementary figures and images for: Trial sequential meta-analysis of laparoscopic versus open pancreaticoduodenectomy: is it the time to stop the randomization?
Source: Surg Endosc. 2022 Oct 17;37(3):1878–89. doi: 10.1007/s00464-022-09660-6 (PMC10017649; doi:10.1007/s00464-022-09660-6)

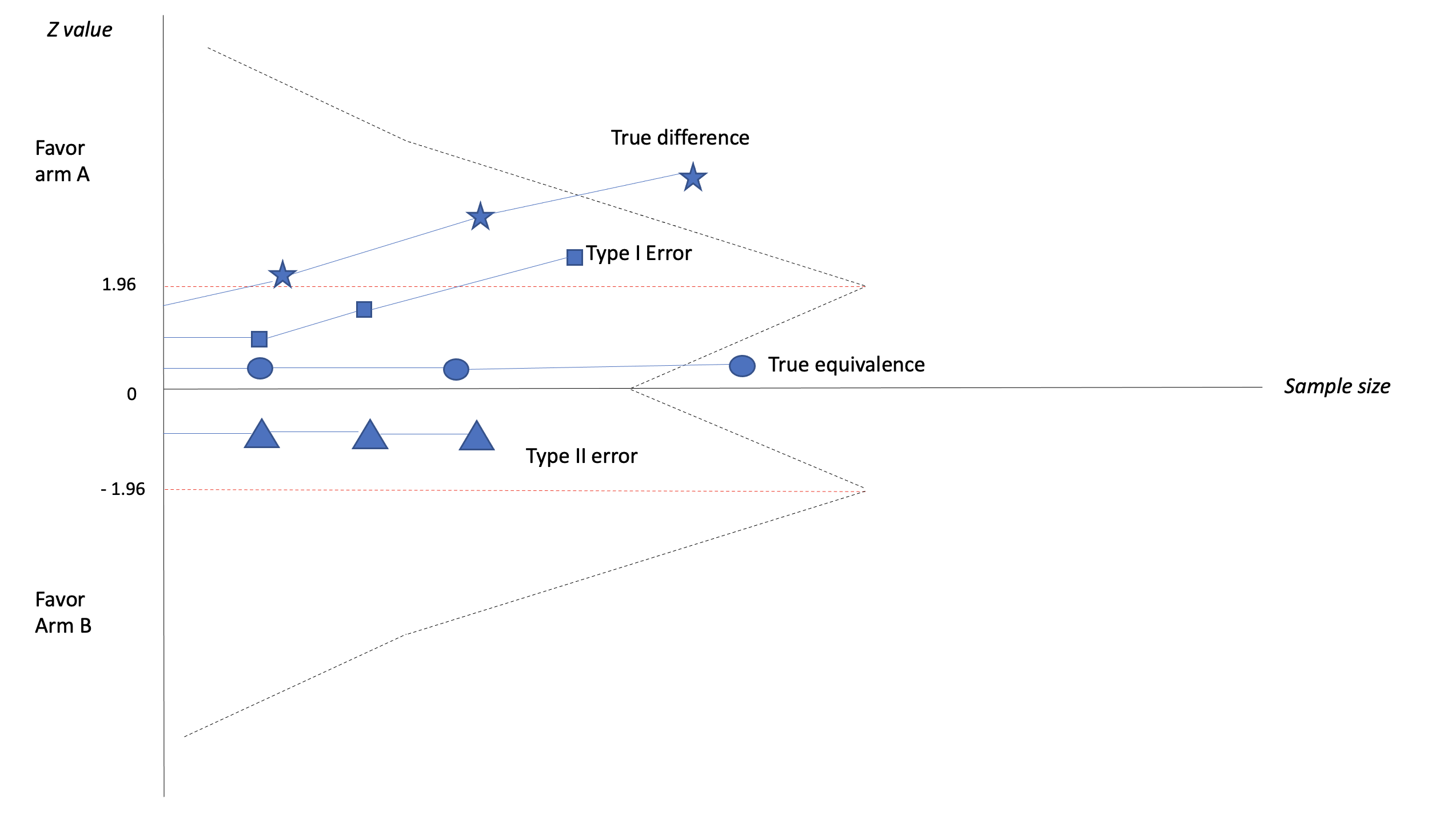

Supplement: Supplementary file 1 — Electronic supplementary material 1 (PNG 150 kb) [file 464_2022_9660_MOESM1_ESM.png]
