# Supplementary material for: Trial sequential meta-analysis of laparoscopic versus open pancreaticoduodenectomy: is it the time to stop the randomization?
Source: Surg Endosc. 2022 Oct 17;37(3):1878–89. doi: 10.1007/s00464-022-09660-6 (PMC10017649; doi:10.1007/s00464-022-09660-6)
Supplement: Supplementary file 2 — Electronic supplementary material 2 (DOCX 18 kb) [file 464_2022_9660_MOESM2_ESM.docx]

**Supplementary Methods**

No language, publication date, or status restrictions were used. A systematic literature search was done through Pubmed, Scopus, the ISI-Web of Science, and Cochrane Central Register of Controlled Trials (CENTRAL), updating the last systematic search. The string used was the following adapted for each database using SR accelerator^xx^: (laparoscop*[tiab] OR ‘‘minimally invasive’’ [tiab] OR ‘‘minimal invasive’’ [tiab] OR hybrid [tiab] OR robot_[tiab] OR telerobotic*[tiab] OR Vinci[tiab] OR daVinci[tiab] OR ‘‘Robotic Surgical Procedures’’[Mesh] OR laparoscopy [MesH]) AND ((pancreatoduodenectom*[tiab] OR pancreaticoduodenectomy [MesH] OR ‘‘Pancreatectomy’’[Mesh] OR pancreaticoduodenectom* [tiab] OR PPPD[tiab] OR pancreatectom*[tiab] OR duodenopancreatectom*[tiab] OR Kausch-Whipple OR Whipple*[tiab] OR ppWhipple*[tiab] OR ‘‘pancreatic head resection’’ [tiab] OR ‘‘Pancreatic Neoplasms/surgery’’[Mesh]) OR ((pancreas[tiab] OR pancreatic*[tiab] OR ‘‘Pancreas’’[Mesh] OR ‘‘Pancreatic Diseases’’[Mesh]) AND (surger*[tiab] OR surgical[tiab] OR operation*[tiab] OR resection*[tiab]))). The last research was performed on 28 January 2022. The related articles were used to broaden the search, and all the abstracts, studies, and citations were reviewed. Also, the references of all the studies included were screened for other potentially relevant studies. SR accelerator was used to manage the literature results. A 2020 PRISMA flowchart was built to report the conclusions reached by the authors. The retrieved studies were first evaluated using the title and abstract to remove the non-relevant records. Thus, the eligibility was established by reading the full text of the remaining articles using the following inclusion and exclusion criteria. The inclusion criteria were: 1) randomized design; 2) LPD as intervention arm; 3) OPD as control arm; 4) extractable postoperative morbidity and mortality, at least. The following exclusion criteria were used: 1) abstracts without a full-text or an unpublished report; 2) studies without original data and reviews or meta-analyses; 3) studies with unextractable data and 4) studies without outcomes of interest. The selection process was carried out in a blinded manner by two different reviewers (C.I. and L.A.). All eligible studies were evaluated in full-text form. When the study met all the inclusion criteria, none of the exclusion was included. Two independent authors (C.I. and A.S.) extracted the data using prefixed standardized data forms. The heterogeneity was evaluated using I^2^ and Cochran's Q statistics.^1^ If I^2^ was <50%, the heterogeneity was considered low-moderate. When I^2^ was ≥50%, the heterogeneity was judged high, and a meta-regression was performed. The heterogeneity was also calculated as diversity (D^2^). The D^2^ is directly constructed to fulfill the RIS calculation. It is not dependent on any "a priori" sampling error estimate. ^2^ The following confounding covariates were considered: age, gender, Body Mass Index (BMI), American Society of Anesthesiology (ASA) score, preoperative stent, the texture of pancreatic remnant, tumor' size, pancreatic adenocarcinoma rate, malignant lesions rate, country, study design (blinded or not), and the number of per capita procedures (< or ≥ 20). If the distribution of confounding covariates changed between two arms for each study, they were reported as RR or MD with an entire 95% CI. The effect of each covariate on the outcome was described using the coefficient with 95% CI. Thus, the coefficient was related to the change in the target event unit of RR or MD. For example, if the coefficient was > 0, an increased RR or MD of the covariate in the LPD group positively modified the RR or MD in the same group. The proportion of heterogeneity explained by covariate was calculated and reported as R^2^. The meta-regression was based on maximum residual likelihood (REML). ^3-4^ The publication bias was evaluated using the Begg and the Egger tests ^5^, and a P-value <0.05 indicated a non-negligible "small-study effect." The statistical analysis was carried out using dedicated packages for STATA v14^®^. TSA was conducted using the Trial Sequential Analysis software.^6^ The funnel plot was produced using Revman version 6.1.

References

1. Higgins JP, Thompson SG. Quantifying heterogeneity in a meta-analysis. Stat Med 2002; 21: 1539-1558.
2. Wetterslev J, Thorlund K, Brok J, Gluud C. Estimating required information size by quantifying diversity in random-effects model meta-analyses. BMC Med Res Methodol. 2009
3. Thompson SG, Sharp SJ.Explaining heterogeneity in meta-analysis: a comparison of methods. Stat Med. 1999;18:2693-2708.
4. Higgins JP and Thompson SG. Controlling the risk from spurious findings from meta-regression. Statistics in Medicine 2004; 23:1663-1682.
5. Egger M, Davey Smith G, Schneider M, et al. Bias in meta-analysis detected by a simple, graphical test. BMJ 1997: 315; 629-634
6. Wetterslev J, Jakobsen JC, Gluud C. Trial Sequential Analysis in systematic reviews with meta-analysis. BMC Med Res Methodol. 2017;17(1):39.
