# Supplementary material for: Trial sequential meta-analysis of laparoscopic versus open pancreaticoduodenectomy: is it the time to stop the randomization?
Source: Surg Endosc. 2022 Oct 17;37(3):1878–89. doi: 10.1007/s00464-022-09660-6 (PMC10017649; doi:10.1007/s00464-022-09660-6)
Supplement: Supplementary file 3 — Electronic supplementary material 3 (DOCX 15 kb) [file 464_2022_9660_MOESM3_ESM.docx]

*Secondary endpoints*

*R1 resection*

The R1 risk was similar between the two groups, with a pooled RR of 0.77 (0.50 to 1.19, 95% CI). The RIS at the current RR was 6,475, suggesting that 5,657 patients should be further randomized before concluding that LPD and OPD are equal without occurring in type II error. The additional RISs calculated for the four scenarios demonstrated that both LPD and OPD did not reduce by 50 % the risk of CR-POPF because the Z-curve is close to RIS (1,093 and 1,742). Still, it has yet crossed the futility boundaries**.** The assumption that LPD or OPD reduced by 25% of the RR could be demonstrated or rejected only cumulating 5,069 or 6,371 randomized patients.

*PPH grades B and C*

PPH grade B/C risk was similar among the two groups, with a RR of 0.84 (0.54 to 1.30; 95% CI) and a RIS of 11,486. Additional 10,668 patients should be randomized before obtaining a credible result about PPH. The additional RISs calculated for the four scenarios demonstrated that: both LPD and OPD did not reduce by 50 % the risk of PPH because the Z-curve is close to RIS (891 and 1406), but it has yet crossed the futility boundaries. The assumption that LPD or OPD reduced by 25% of the RR could be demonstrated or rejected only cumulating 4,101 or 5,131 randomized patients.

*DGE grades B and C*

The risk of DGE was similar among the two groups, with a RR of 0.95 (0.59 to 1.53; 95% CI). The RIS was 253,461 at the current RR. Additional 252,643 patients should be randomized before accepting the equivalence hypothesis of the two approaches. The additional RISs calculated for the four scenarios demonstrated that both LPD and OPD did not reduce by 50 % the risk of CR-POPF because the Z-curve is close to RIS (1,152 and 1,748). Still, it has yet crossed the futility boundaries. The assumption that LPD or OPD reduced by 25% of the RR could be demonstrated or rejected only cumulating 5,269 or 6,427 randomized patients.

*Biliary Fistula*

The biliary fistula risk was similar among the two groups, with a RR of 1.33 (0.73 to 2.41; 95% CI) and a RIS of 8,183**.** Additional 7,365 patients should be randomized before accepting the equivalence hypothesis of the two approaches. The other RIS to demonstrate that LPD reduced by 50 % the risk of the biliary fistula was 1,934. The Z-curve is close to the futility boundaries. The additional RIS shows that OPD reduced by 50 % the risk of the biliary fistula was 3,144. Further, 2,326 should be randomized before confirming or rejecting the null hypothesis. The assumption that LPD or OPD reduced by 25% of the RR could be demonstrated or rejected only cumulating 5,269 or 6,427 randomized patients**.**

*Reoperation rate*

The risk reoperation was similar among the two groups, with a pooled RR of 0.92 (0.42 to 2.01, 95% CI). The RIS at the current RR was 47,178, suggesting that -43,360 patients should be further randomized before concluding that LPD and OPD are equal without occurring in type II error. The RIS demonstrates that LPD was reduced by 50 % the reoperation risk was 1,934. The Z-curve is close to the futility boundaries. RIS value of 3,708 was obtained to show or reject the hypothesis that OPD could reduce by 50% the RR of mortality rates. The RISs required to get credible information about LPD or OPD's ability to lower 25% the mortality was 10,492 and 13,538.

*Readmission rate*

The risk of readmission was similar among the two groups, with a RR of 1.12 (0.66 to 1.90; 95% CI) and a RIS of 22,032. Additional 21,214 patients should be randomized before obtaining a credible result about readmission. The RIS demonstrates that LPD was reduced by 50 % the reoperation risk was 1,934. At this value, the Z-curve crossed the futility boundaries. A similar RIS (2,607) value was obtained, assuming that OPD could reduce by 50% the RR of mortality rates. At this value, the Z-curve is close to futility boundaries. The assumption that LPD or OPD reduced by 25% of the RR could be demonstrated or rejected only cumulating 7,465 or 9,456 randomized patients.

*Operative time*

The operative time was longer in the LPD than in the OPD group, with an MD of 63 minutes (9.54 to 116.89, 95 CI). The RIS 1,273 was, and Z-curve crossed the conventional boundary, and it is near to benefit edge in favor of OPD. The additional RISs demonstrated that the non-inferiority of the two techniques, assuming a difference of 10 minutes and 30 minutes, should be shown only after 39,974 and 4,269 randomized patients. On the contrary, a clear superiority of OPD of about 120 minutes or 90 minutes was excluded because the RISs were 374 and 599. At these values, the Z-curves have yet to cross the futility boundaries.

*Lymph nodes harvested*

The mean number of lymph nodes harvested was similar (MD -0.98; -2.40 to 0.44, 95 CI). The RIS was 3,515, and additional 2,693 patients should be randomized before obtaining a credible result about this outcome. The additional RISs demonstrated that the non-inferiority of the two techniques, assuming a difference of 3 lymph nodes, is shown already after 302 randomized patients. The Z-curve crossed the futility boundaries. A non-inferiority, considering a difference of 1 lymph node, should be demonstrated only after 2660 randomized patients.

*Length of stay*

The LOS was shorter in the LPD than in the OPD group, with an MD of -1.76 days (-3.32 to -0.21, 95 CI). The RIS 1,297 was, and Z-curve crossed the conventional boundary, and it is near to benefit edge in favor of LPD. The additional RISs demonstrated that the non-inferiority of the two techniques, assuming a difference of 3 days, is shown already after randomized 354 patients. A non-inferiority, considering a difference of 1 day, should be demonstrated only after 3,180 randomized patients.
