# Supplementary material for: Trial sequential meta-analysis of laparoscopic versus open pancreaticoduodenectomy: is it the time to stop the randomization?
Source: Surg Endosc. 2022 Oct 17;37(3):1878–89. doi: 10.1007/s00464-022-09660-6 (PMC10017649; doi:10.1007/s00464-022-09660-6)
Supplement: Supplementary file 4 — Electronic supplementary material 4 (DOCX 36 kb) [file 464_2022_9660_MOESM4_ESM.docx]

| Covariates | Number of studies | RR or SMD (95% CI) | P-value | Heterogeneity |
| --- | --- | --- | --- | --- |
| Age | 4 | 0.02 (-0.21 to 0.19) | 0.829 | 36.3 |
| Male gender | 4 | 085 (0.76 to 0.96) | 0.006 | 0 |
| BMI | 4 | 0.02 (-0.31 to 0.26) | 0.866 | 0 |
| ASA I/II | 4 | 0.98 (0.92 to 1.01) | 0.593 | 0 |
| Preoperative stent | 3 | 0.92 (0.74 to 1.14) | 0.444 | 0 |
| Soft pancreas (RR) | 3 | 0.85 (0.65 to 1.12) | 0.249 | 63 |
| Tumor size (MD, cm) | 4 | -0.02 (-0.97 to 0.94) | 0.971 | 0 |
| Pancreatic adenocarcinoma (RR) | 4 | 0.82 (0.60 to 1.15) | 0.259 | 50 |
| Malignant lesions (RR) | 4 | 0.99 (0.92 to 1.07) | 0.804 | 0 |

**Supplementary Table 1. Covariates potentially influencing the outcomes with high heterogeneity. In the table the distribution among the two groups was reported.**

**Legend =** RR risk ratio; SMD= standardized mean difference; BMI= Body Mass Index; ASA= American Society of Anesthesiology; PDAC= Pancreatic ductal adenocarcinoma.

**Supplementary Table 2. Covariates potentially influence the mortality rate**

| Covariates * | Number of studies | Coeff. ** | P-value after 1000 Montecarlo permutation | Adjusted R^2^ |
| --- | --- | --- | --- | --- |
| Age (MD, years) | 4 | -5.9 (-42.6 to 30) | 0.556 | <1 |
| Male gender (RR) | 4 | -3.8 (-69 to 61.7) | 0.827 | <1 |
| BMI (MD, kg/m^2^) | 4 | 0.5 (-4.1 to 4.9) | 0.684 | <1 |
| ASA I/II (RR) | 4 | -0.2 (-143.4 to 142.9) | 0.996 | <1 |
| Preoperative stent (RR) | 3 | -12.6 (-295.3 to 269) | 0.670 | <1 |
| Soft pancreas (RR) | 3 | -7.5 (-135.4 to 120.4) | 0.592 | <1 |
| Tumor size (MD, cm) | 4 | 20.2 (-117.4 to 157.9) | 0.592 | <1 |
| Pancreatic adenocarcinoma (RR) | 4 | -1.7 (-16.4 to 13.1) | 0.674 | <1 |
| Malignant lesions (RR) | 4 | 38.2 (6.4 to 69.9) | 0.035 | 100 |
| Country (Eastern vs. Western) | 4 | 2.8 (-11.3 to 16.9) | 0.484 | <1 |
| Design (open vs. blinded) | 4 | 3.1 (-10.8 to 17.1) | 0.436 | <1 |
| Number of per-capita procedure (< 20 vs. > 20) | 4 | -6.1 (-11.1 to -1.1) | 0.035 | 100 |

**Legend**: *= The values were reported as mean difference (MD) or risk ratio between LPD to OPD; the coefficient indicates the positive or negative change of RR; BMI= Body Mass Index; ASA= American Society of Anesthesiology; PDAC= Pancreatic ductal adenocarcinoma.

**Supplementary Table 3. Covariates potentially influence the major morbidity rate**

| Covariates * | Number of studies | Coeff. ** | P-value after 1000 Montecarlo permutation | Adjusted R^2^ |
| --- | --- | --- | --- | --- |
| Age (MD, years) | 4 | -1.8 (-7.3 to 3.7) | 0.293 | <1 |
| Male gender (RR) | 4 | -1.9 (-7.9 to 4.1) | 0.308 | <1 |
| BMI (MD, kg/m^2^) | 4 | 0.1 (-0.9 to 1.1) | 0.760 | <1 |
| ASA I/II (RR) | 4 | 3.5 (-10.7 to 17.7) | 0.401 | <1 |
| Preoperative stent (RR) | 3 | -2.2 (-41.9 to 37.5) | 0.611 | <1 |
| Soft pancreas (RR) | 3 | -1.6 (-13.8 to 10.5) | 0.335 | <1 |
| Tumor size (MD, cm) | 4 | 3.5 (-15.3 to 22.3) | 0.507 | <1 |
| Pancreatic adenocarcinoma (RR) | 4 | -0.6 (-2.9 to 1.7) | 0.398 | <1 |
| Malignant lesions (RR) | 4 | 2.4 (-9.9 to 14.8) | 0.481 | <1 |
| Country (Eastern vs. Western) | 4 | -0.1 (-2.2 to 1.9) | 0.787 | <1 |
| Design (open vs. blinded) | 4 | 0.7 (-1.1 to 2.5) | 0.218 | <1 |
| Number of per-capita procedure (< 20 vs. > 20) | 4 | -0.3 (-2.5 to 1.8) | 0.587 | <1 |

**Legend**: *= The values were reported as mean difference (MD) or risk ratio between LPD to OPD; the coefficient indicates the change of RR of LPD vs OPD comparison; BMI= Body Mass Index; ASA= American Society of Anesthesiology; PDAC= Pancreatic ductal adenocarcinoma.

| Covariates * | Number of studies | Coeff. ** | P-value after 1000 Montecarlo permutation | Adjusted R^2^ |
| --- | --- | --- | --- | --- |
| Age (MD, years) | 4 | -1.2 (-7.4 to 4.9) | 0.476 | <1 |
| Male gender (RR) | 4 | -1.3 (-13.3 to 10.7) | 0.687 | <1 |
| BMI (MD, kg/m^2^) | 4 | 0.1 (-0.7 to 0.9) | 0.616 | <1 |
| ASA I/II (RR) | 4 | 1.2 (-24.2 to 26.8) | 0.851 | <1 |
| Preoperative stent (RR) | 3 | -2.4 (-43.7 to 38.9) | 0.595 | <1 |
| Soft pancreas (RR) | 3 | -1.8 (-22.2 to 18.6) | 0.469 | <1 |
| Tumor size (MD, cm) | 4 | 4.4 (-21.5 to 30.3) | 0.538 | <1 |
| Pancreatic adenocarcinoma (RR) | 4 | -0.4 (-2.9 to 2.1) | 0.588 | <1 |
| Malignant lesions (RR) | 4 | 5.4 (-5.3 to 16.2) | 0.162 | 100 |
| Country (Eastern vs. Western) | 4 | 0.4 (-1.9 to 2.6) | 0.523 | 7 |
| Design (open vs. blinded) | 4 | 0.7 (1.8 to 3.1) | 0.369 | <1 |
| Number of per-capita procedure (< 20 vs. > 20) | 4 | -0.8 (-2.5 to 0.9) | 0.174 | 100 |

**Supplementary Table 4. Covariates potentially influence DGE.**

**Legend**: *= The values were reported as mean difference (MD) or risk ratio between LPD to OPD; the coefficient indicates the change of RR of LPD vs OPD comparison; BMI= Body Mass Index; ASA= American Society of Anesthesiology; PDAC= Pancreatic ductal adenocarcinoma.

**Supplementary Table 5. Covariates potentially influence operative time**

| Covariates * | Number of studies | Coeff. ** | P-value after 1000 Montecarlo permutation | Adjusted R^2^ |
| --- | --- | --- | --- | --- |
| Age (MD, years) | 4 | -184.2 (-467.4 to 98.9) | 0.107 | 71 |
| Male gender (RR) | 4 | -135.5 (-1073.9 to 802.9) | 0.598 | <1 |
| BMI (MD, kg/m^2^) | 4 | 14.9 (-40.5 to 70.4) | 0.366 | 9 |
| ASA I/II (RR) | 4 | 302.6 (-1755.5 to 2360.8) | 0.592 | <1 |
| Preoperative stent (RR) | 3 | -391.4 (-635.4 to -147.5) | 0.031 | 100 |
| Soft pancreas (RR) | 3 | -147.1 (-253.1 to -41.2) | 0.036 | 100 |
| Tumor size (MD, cm) | 4 | -9.2 (-2280.3 to 2261.9) | 0.988 | <1 |
| Pancreatic adenocarcinoma (RR) | 4 | -63.1 (-208.2 to 82.2) | 0.203 | 45 |
| Malignant lesions (RR) | 4 | 409.5 (-724.1 to 1543.3) | 0.260 | 40 |
| Country (Eastern vs. Western) | 4 | 14.3 (-237.9 to 266.5) | 0.830 | <1 |
| Design (open vs. blinded) | 4 | 85.1 (32.1 to 137.1) | 0.120 | <1 |
| Number of per-capita procedure (< 20 vs. > 20) | 4 | 64.2 (-278.5 to 150.2) | 0.327 | 27 |

**Legend**: *= The values were reported as mean difference (MD) or risk ratio between LPD to OPD; the coefficient indicates the change of MD of LPD vs OPD comparison; BMI= Body Mass Index; ASA= American Society of Anesthesiology; PDAC= Pancreatic ductal adenocarcinoma.

**Supplementary Table 5. Covariates potentially influence lymph nodes harvested**

| Covariates * | Number of studies | Coeff. ** | P-value after 1000 Montecarlo permutation | Adjusted R^2^ |
| --- | --- | --- | --- | --- |
| Age (MD, years) | 4 | 13.7 (-152.7 to 180.1) | 0.757 | <1 |
| Male gender (RR) | 4 | -28.1 (-247.1 to 190.8) | 0.636 | <1 |
| BMI (MD, kg/m^2^) | 4 | -1.6 (-16.1 to 12.8) | 0.678 | <1 |
| ASA I/II (RR) | 4 | 72.3 (-491.1 to 635.6) | 0.636 | <1 |
| Preoperative stent (RR) | 3 | 8.3 (-1181.1 to 1197.7) | 0.943 | <1 |
| Soft pancreas (RR) | 3 | -11.3 (-537.8 to 515.1) | 0.830 | 0 |
| Tumor size (MD, cm) | 4 | 53.4 (-361.9 to 468.8) | 0.636 | <1 |
| Pancreatic adenocarcinoma (RR) | 4 | 5.7 (-50.7 to 62.1) | 0.706 | <1 |
| Malignant lesions (RR) | 4 | 27.8 (-472.7 to 528.4) | 0.833 | <1 |
| Country (Eastern vs. Western) | 4 | -6.8 (-67.3 to 53.7) | 0.677 | <1 |
| Design (open vs. blinded) | 4 | 2.4 (-87.5 to 92.4) | 0.918 | <1 |
| Number of per-capita procedure (< 20 vs. > 20) | 4 | -2.4 (-93.3 to 88.5) | 0.920 | <1 |

**Legend**: *= The values were reported as mean difference (MD) or risk ratio between LPD to OPD; the coefficient indicates the change of MD of LPD vs OPD comparison; BMI= Body Mass Index; ASA= American Society of Anesthesiology; PDAC= Pancreatic ductal adenocarcinoma.
